# Thrombocytopenia-Absent Radius Syndrome: Descriptions of Three New Cases and a Novel Splicing Variant in *RBM8A* That Expands the Spectrum of Null Alleles

**DOI:** 10.3390/ijms23179621

**Published:** 2022-08-25

**Authors:** Catarina Monteiro, Ana Gonçalves, Jorge Oliveira, Ramon Salvado, Jorge Tomaz, Sara Morais, Margarida Lima, Rosário Santos

**Affiliations:** 1 Unidade de Genética Molecular, Centro de Genética Médica Doutor Jacinto Magalhães, Centro Hospitalar Universitário do Porto, 4099-028 Porto, Portugal; 2 Serviço de Hematologia Clínica, Unidade de Trombose e Hemostase, Centro Hospitalar Universitário do Porto, 4099-001 Porto, Portugal; 3 Unidade Multidisciplinar de Investigação Biomédica (UMIB), Instituto de Ciências Biomédicas Abel Salazar (ICBAS) e Laboratório Para a Investigação Integrativa e Translacional em Saúde Populacional (ITR), Universidade do Porto, 4050-313 Porto, Portugal; 4 Serviço de Imunohemoterapia, Centro Hospitalar Universitário de Coimbra, 3000-075 Coimbra, Portugal

**Keywords:** thrombocytopenia-absent radius (TAR) syndrome, *RBM8A*, 1q21 microdeletion, neonatal thrombocytopenia, null allele

## Abstract

Thrombocytopenia-absent radius (TAR) syndrome is a rare congenital disorder characterized by the bilateral absence of the radius and thrombocytopenia, and sometimes by other skeletal, gastrointestinal, cardiac, and renal abnormalities. The underlying genetic defect is usually the compound inheritance of a microdeletion in 1q21.1 (null allele) and a low-frequency, non-coding single nucleotide variant (SNV) in the *RBM8A* gene (hypomorphic allele). We report three new cases from two unrelated families. The two siblings presented the common genotype, namely the compound heterozygosity for a 1q21.1 microdeletion and the hypomorphic SNV c.-21G>A in *RBM8A*, whereas the third, unrelated patient presented a rare genotype comprised by two *RBM8A* variants: c.-21G>A (hypomorphic allele) and a novel pathogenic variant, c.343-2A>G (null allele). Of the eight documented *RBM8A* variants identified in TAR syndrome patients, four have hypomorphic expression and four behave as null alleles. The present report expands the *RBM8A* null allele spectrum and corroborates the particularities of *RBM8A* involvement in TAR syndrome pathogenesis.

## 1. Introduction

Thrombocytopenia-absent radius syndrome (TAR (MIM #274000)) is a well-characterized congenital disorder defined as hypomegakaryocytic thrombocytopenia and bilateral radial aplasia in the presence of both thumbs [1,2,3]. With an incidence of 1:100.000–1:200.000 live births and affecting both genders, TAR syndrome was first described by Gross et al. and Shaw and Oliver in 1959, and defined as a syndrome by Hall years later [1,2,3,4,5].

Besides radial aplasia, TAR syndrome may comprise other skeletal anomalies, such as shortening or aplasia of the ulna and/or humerus, lower limb malformations, hip luxation, and upper limb phocomelia. Extra-skeletal abnormalities include cardiac and renal anomalies and an intolerance to cow milk [2,5]. Dysmorphic features and macrocephaly can also be found in more severe cases [2].

The pathophysiology of thrombocytopenia is poorly understood; the bone marrow is hypercellular, with low, absent, or immature megakaryocytes (MGK) [5]. Thrombocytopenia is symptomatic in over 90% of the cases, with bleeding episodes; the platelet (PLT) count may improve and bleeding may diminish with age [3,4,6].

To uncover the underlying genetic basis of the syndrome, Klopocki et al. applied microarray-based comparative genomic hybridization (array CGH) to a cohort of patients with a 1q21.1 microdeletion, and they identified a minimal critical region of ~200 kb, encompassing eleven known genes [4]. However, as the microdeletion was also present in some unaffected family members, the authors proposed the existence of an additional modifier.

The modifying allele remained elusive until 2012, when Albers et al. first described the involvement of low-frequency single nucleotide variants (SNVs) in the regulatory regions of the *RBM8A* gene [7]. Together with the 1q21.1 microdeletion, most patients carried either the noncoding SNV c.-21G>A in the 5′ untranslated region (UTR) or the intronic SNV c.67+32G>C. Moreover, two patients without the microdeletion were found to carry the *RBM8A* 5′UTR SNV and an allelic loss-of-function variant (c.207_208insAGCG or c.487C>T). These data suggested that the genetic basis of TAR syndrome is compound heterozygosity for a noncoding, hypomorphic SNV and a rare null allele, both involving *RBM8A* [3,7]. Recently, Boussion et al. described four other pathogenic *RBM8A* variants amongst a cohort of 26 patients [8].

The *RBM8A* gene encodes the RNA-binding motif protein A, also designated the Y14 protein, which is a component of the exon junction complex (EJC), involved in numerous essential cellular functions [9,10,11]. Given this housekeeping role of the EJC, *RBM8A* is widely expressed, including in MGK and osteoblasts [9,10,12,13]. Functional studies have shown that Y14 levels are significantly lower in the PLTs of TAR syndrome patients, suggesting that here, *RBM8A* variants decrease protein expression to levels below a critical threshold [3,9,12].

We report three additional TAR syndrome patients, from two unrelated families, diagnosed at birth and later reassessed following comprehensive genetic studies that revealed the involvement of the *RBM8A* gene. Besides corroborating the genotypic signature of the syndrome, this work describes a novel pathogenic variant, further expanding the mutational profile of *RMB8A* null alleles.

## 2. Detailed Case Description

### 2.1. Case Presentation

#### 2.1.1. Family 1

Two affected siblings (F1-II.1 and F1-II.2) were born five years apart to healthy consanguineous parents with no intercurrences during either pregnancy. Case F1-II.1 was a first gestation female. At birth, she presented with upper limb malformations and generalized petechiae. X-rays showed the bilateral absence of the radii and ulna hypoplasia, and the PLT count was 10 × 10^9^/L. She was admitted to a neonatology unit prior to invasive procedures for several PLT transfusions to treat bleeding symptoms. At that time, her cardiac, cerebral, and abdominal ultrasounds were normal, but her bilateral congenital hip luxation was identified and corrected nine months later under PLT transfusion. At 18 years of age, she presented PLT counts of 145 × 10^9^/L. The younger female sibling (F1-II.2) was born with the bilateral absence of the radii, severe thrombocytopenia (18 × 10^9^/L), cardiac malformations (small atrial septal defect), strabismus, and transient leukemoid reaction, with white blood cell counts of 52 × 10^9^/L. No other skeletal alterations were observed. She underwent PLT transfusions for an episode of epistaxis and surgeries for the correction of strabismus. At age 13, her PLT counts were 113 × 10^9^/L.

#### 2.1.2. Family 2

Case F2-II.1 is a female neonate born to healthy non-consanguineous parents following a pregnancy without intercurrences. She had petechiae, ecchymosis, and bilateral deformities with the radial deviation of both upper limbs. There were no facial dysmorphic features, nor were there cerebral, cardiac, or renal alterations. X-rays confirmed the bilateral absence of the radii with the presence of both thumbs and minor bilateral ulna hypoplasia. She had PLT counts of 20 × 10^9^/L, rare megakaryocytes in the bone marrow, and a normal karyotype [14]. She maintained thrombocytopenia with platelet counts of 10 to 17 × 10^9^/L, and she had three transient episodes of leukemoid reactions (30 to 40 × 10^9^/L) during infectious episodes. Poor weight gain, diarrhea, and cow milk intolerance were identified. Since her 18 months of age, there has been a progressive increase in the PLT counts and suitable ponderal, mental, and psychomotor development. Presently, at age 26 years, she has an average PLT count of 90 × 10^9^/L. The improvement of the thrombocytopenia enabled two orthopedic surgeries in the upper limbs.

### 2.2. Genetic Studies

#### 2.2.1. Identification of a 1q21.1 Microdeletion and a Low-Frequency SNV in *RBM8A*

In both siblings of family 1, MLPA screening detected a 1q21.1 microdeletion of approximately 302.52kb (rsa 1q21.1 (HFE2, PEX11B, CD160)x1), encompassing 14 MIM genes: *HFE2*, *TXNIP*, *POLR3GL*, *ANKRD34A*, *LIX1L*, *RBM8A*, *PEX11B*, *ITGA10*, *ANKRD35*, *PIAS3*, *NUDT17*, *POLR3C*, *RNF115*, and *CD160* (data not shown).

Targeted Sanger sequencing of the 5′ UTR of *RBM8A* (NM_005105.4; LRG_574t1) detected the known low-frequency SNV c.-21G>A, thereby establishing the molecular diagnosis of TAR syndrome. Compound heterozygosity of the microdeletion and a 5′ UTR SNV is the most common underlying genotype of TAR syndrome [3,4,6,12,15,16,17]. The parents were unavailable for segregation analysis, but the two variants detected in these siblings conforms to this common genotype, as does their classical presentation of TAR syndrome.

#### 2.2.2. Identification of a Low-Frequency SNV and a Novel Splicing Variant in *RBM8A*

In family 2 (case F2-II.1), no 1q21.1 microdeletion was detected by MLPA analysis. High-throughput sequencing revealed two heterozygous *RBM8A* variants: the known 5′ UTR SNV c.-21G>A (as in family 1) and the undocumented intronic variant c.343-2A>G, coincident with a canonical acceptor splice site. Both variants were confirmed by Sanger sequencing and no other pathogenic or likely pathogenic variants were identified. Segregation analysis revealed that the 5′ UTR variant was inherited maternally. The father was unavailable for study, therefore it was not ascertained whether the novel variant was inherited paternally or had occurred de novo. Nevertheless, compound heterozygosity for these variants is highly likely and would explain the underlying genetic cause of TAR syndrome in the patient.

**Figure 1:**
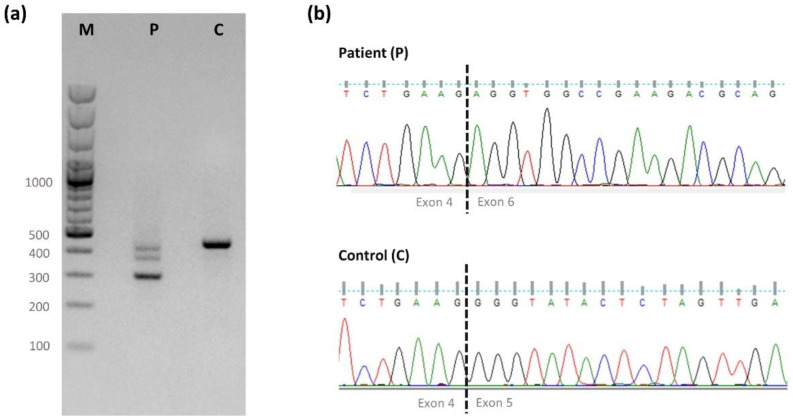
(**a**) Amplification products of *RBM8A* cDNA exons 2 to 6 (F2-II.1.) The control sample (C) shows a normal-sized fragment (~460 bp), as expected for the region encompassing exons 2 to 6. The patient (P) presents a faint, normal-sized fragment and a predominantly shorter fragment (~320 bp), consistent with the absence of exon 5. The residual intermediate-sized fragment reflects heteroduplex formation (verified by Sanger sequencing; data not shown). (**b**) Partial electropherograms showing the exon 4/exon 6 junction sequence in the patient’s aberrant fragment and the normal exon 4/exon 5 junction sequence in the control. M—size markers: GeneRuler 100 bp Plus DNA ladder (Thermo Fisher Scientific, Waltham, MA, USA).

As bioinformatic analysis strongly suggested that the novel variant in intron 4 affects pre-mRNA processing, *RBM8A* cDNA analysis was carried out (Figure 1). Besides a residual amount of wildtype transcript, a predominant out-of-frame transcript was observed, corresponding to the skipping of exon 5 (r.343_479del), and predictably resulting in a non-functional or no-protein product (p.(Gly115Argfs*30)).

## 3. Discussion

Thrombocytopenia-absent radius is a syndromic bleeding disorder characterized by low PLT counts in association with the bilateral absence of the radii. Previous work has shown that TAR syndrome is a complex genetic disorder caused by the compound inheritance of a rare null allele and a low frequency hypomorphic noncoding variant in the *RBM8A* gene [3,7,8,18]. It was demonstrated that this combination, where one copy of the gene is absent or non-functional and the expression of the other allele is downregulated, culminates in the diminished expression of the encoded Y14 protein, which is a constituent of the EJC [3,4,7,8].

**Figure 2:**
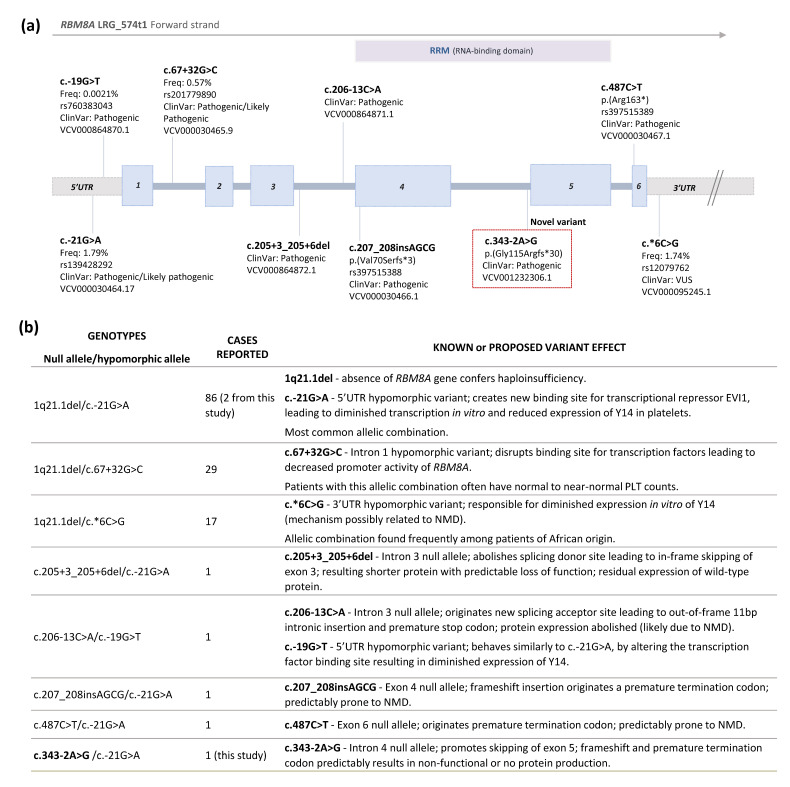
(**a**) Schematic representation of the *RBM8A* gene and the localization of TAR syndrome associated variants classified as pathogenic/likely pathogenic, as documented in the literature. The novel variant identified in this work is highlighted in a box. The nomenclature is used according to HGVS, using Reference Sequence NM_005105.4, LRG_574t1. When available, frequencies in the gnomAD database and dbSNP identification references are shown. Blue boxes—exons; grey boxes—UTRs; RRM—RNA recognition motif; VUS—variant of uncertain significance. (**b**) Summary of documented TAR syndrome genotypes and known/predicted effects of the variants. NMD—nonsense-mediated mRNA decay. Source [2,3,6,8,12,15,16,17,18,19,20,21,22,23,24,25,26].

The identification of novel *RBM8A* variants and their implication in the pathophysiology of TAR syndrome is of extreme value, as it has been demonstrated that specific clinical features might be associated with specific variants and their location in the gene [8,18]. It has been seen, for example, that the 5′ UTR SNP c.-21G>A correlates with a lower PLT count and red blood cell production defects, which could normalize over the years, whereas the intronic SNP c.67+32G>C seems to be related with higher PLT counts, but not with red blood cells anomalies [19]. A search in the literature for TAR syndrome cases that were genetically characterized revealed over 130 cases and seven different genotypes, as summarized in Figure 2b [6,8,12,15,16,17,18,19,20,21,22,23,24,25,26].

We report a common genetic constitution of TAR syndrome in two siblings (family 1), increasing the total number of reported cases with the 1q21.1del/c.-21G>A genotype to 86, and one rare case (family 2) with no 1q21.1 microdeletion, but where two *RBM8A* variants are implicated as causative.

In family 2 (case F2-II.1), besides the known hypomorphic SNV c.-21G>A, the intronic variant c.343-2A>G was identified, with in silico analysis predicting an effect on pre-mRNA processing. According to the American College of Medical Genetics and Genomics (ACMG) criteria, this variant was classified as pathogenic (PVS1, PS3, PM2, and PP3). We found that the nucleotide substitution promoted skipping of the entire exon 5, predictably leading to a reading-frame shift and, consequently, a premature termination codon (p.(Gly115Argfs*30)). As this variant affects a canonical splice sequence, mutation leakage is highly unlikely, thereby originating a null allele. Indeed, in a classical TAR patient with a similar genotypic constitution (c.-19G>T/c.206-13C>A), expression and functional studies have demonstrated the abolishment of Y14 production from an allele with an out-of-frame splicing variant in intron 3 (c.206-13C>A) [8]. The residual amount of normal transcript observed in our patient is believed to correspond to the reduced expression from the hypomorphic allele.

As further variants are described in the *RBM8A* gene, it becomes increasingly evident that their type and location may dictate the clinical variability and severity seen in TAR syndrome patients [8,20,26]. We contribute with the description of a further variant that behaves as a null allele, where clinical presentation resembles that of the severe forms often seen in classical cases carrying the common 1q21.1 microdeletion, thereby providing further evidence that TAR syndrome is dictated essentially by the *RBM8A* gene.

Additional studies are required to address the quantitative and qualitative expression of Y14, further define genotype-phenotype correlations, and improve our understanding of how Y14 insufficiency explains the unique skeletal, haematological, and other features of TAR syndrome.

## 4. Materials and Methods

Peripheral blood samples were collected from the patients following informed consent from the parents. Genomic DNA was extracted using the EZ1 Advanced XL DNA blood kit with BioRobotEZ1 (Qiagen Inc., Valencia, CA, USA), according to the manufacturer’s instructions. DNA concentration was determined by spectrophotometry (NanoDrop, Wilmington, DE, USA).

As the most common genetic defect associated with TAR syndrome is a proximal microdeletion in 1q21.1, initially, all three patients were screened by multiplex ligation-dependent probe amplification (MLPA), with probes targeting five regions of the TAR syndrome microdeletion (Supplementary Material S1). Variant screening in the *RBM8A* gene was carried out by conventional Sanger sequencing (cases F1-II.1 and F1-II.2) and by next-generation sequencing (NGS) using a commercial gene panel for haematology (case F2-II.1) (Supplementary Material S1).

### 4.1. Identification of RBM8A Genomic Variants

In family 1, cases II.1 and II.2, screening for the known noncoding SNVs in the 5′ UTR of the *RBM8A* gene was carried out by Sanger sequencing, using custom-designed primers (Table S1). The amplicons were purified with ExoStar 1-stepTM (Illustre ExoProStar Enzymatic PCR and Sequence Reaction clean-up kit (GE Healthcare Life Sciences, Buckinghamshire, UK). Sequencing was carried out using a BigDyeTM Terminator Cycle Sequencing Kit V3 (Thermo Fisher Scientific, Waltham, MA, USA) and processed on an ABI 3130xl genetic analyzer. Electropherograms were analyzed using SeqScape V2.5 software (Thermo Fisher Scientific, Waltham, MA, USA).

For family 2, case II.1, high-throughput sequencing was performed on an Ion GeneStudio S5 sequencer using a commercial panel for haematology targeting 394 genes (Ion AmpliseqTM Hematology Research Panel, Thermo Fisher Scientific, Waltham, MA, USA) and filtering for low-frequency variants (<1%). Given the clinical suspicion, the binary alignment map (BAM) file was meticulously inspected at the *RBM8A* locus using Alamut software (Alamut^TM^ Visual Plus, version 1.5.1, SOPHiA GENETICS SA, Saint-Sulpice, Switzerland) to search for further causative variants. The identified variants were confirmed by Sanger sequencing, as described above, using custom-designed primers (Table S1). HGVS nomenclature was used according to the *RBM8A* reference sequence NM_005105.4; LRG_574t1.

### 4.2. Characterization of a Novel Splice-Site Variant

RNA from patient F2.II.1 and from controls was extracted from peripheral blood using a PerfectPure RNA Blood Kit (5 PRIME, Hamburg, Germany) according to the manufacturer’s protocol. The resulting purified RNA was converted to cDNA following the protocol for the Reverse Transcription SuperScript IV VILO Master MIX with ezDNA (Thermo Fisher Scientific, Waltham, MA, USA). The cDNA region corresponding to *RBM8A* exons 2 to 6 was amplified using custom-designed primers (Table S1). The amplification products were separated on a 1.5% LE agarose gel (GRiSP Research Solutions, Porto, Portugal), excised, purified with ExoStar 1-stepTM, and sequenced as described above. The sequencing results were analyzed with FinchTV 1.4 software and compared to normal controls.

## Data Availability

All data relevant to the study are included in the main text or in the Supplementary Material. Additional supporting data can be provided upon request. The novel variant was submitted to the ClinVar database and is classified as Pathogenic, with the Variation ID 1232306 and Accession Number VCV001232306.2.

## Supplementary Materials

The following supporting information can be downloaded at https://www.mdpi.com/article/10.3390/ijms23179621/s1.
