# Supplementary material for: Thrombocytopenia-Absent Radius Syndrome: Descriptions of Three New Cases and a Novel Splicing Variant in RBM8A That Expands the Spectrum of Null Alleles
Source: Int J Mol Sci. 2022 Aug 25;23(17):9621. doi: 10.3390/ijms23179621 (PMC9455669; doi:10.3390/ijms23179621)
Supplement: Supplementary file 1 [file ijms-23-09621-s001.zip › ijms-1877940-supplementary.pdf]

SUPPLEMENTARY MATERIAL S1

1. Detailed Methodology

Analysis of 1q21.1 locus by MLPA

For all patient and control samples, 150ng gDNA was used, with SALSA MLPA probemix P297-C1 (MRC Holland, The Netherlands), containing probes targeting 5 regions of the TAR syndrome microdeletion*, following the manufacturer's protocol. PCR products were resolved on an ABI 3130xl Genetic Analyser (Applied Biosystems, Foster City, CA, USA) and data was analysed resorting to GeneMarker Software V1.5 (SoftGenetics, State College, PA, USA).

*Probes for TAR syndrome microdeletion: *PDE4DIP* 05712-L05712; *HFE2* 08387-L08241; *PEX11B* 08398-L08252; *PEX11B* 08405-L08259; *CD160* 08373-L08227.

Identification of RBM8A genomic variants

In Family 1, cases II.1 and II.2, screening for the known noncoding SNVs in the 5'UTR of the *RBM8A* gene was carried out by Sanger sequencing, using custom designed primers (Table S1). Amplicons were purified with ExoStar 1-step™ (Illustre ExoProStar Enzymatic PCR and Sequence Reaction clean-up kit, GE Healthcare Life Sciences, Buckinghamshire, UK). Sequencing was carried out using BigDye™ Terminator Cycle Sequencing Kit V3 (Thermo Fisher Scientific, Massachusetts, USA) and processed on an ABI 3130xl Genetic Analyzer. Electropherograms were analysed using SeqScape V2.5 Software (Thermo Fisher Scientific, Massachusetts, USA).

For Family 2, case II.1, high-throughput sequencing was performed on an Ion GeneStudio S5 sequencer using a commercial panel for haematology targeting 394 genes (Ion Ampliseq™ Hematology Research Panel, Thermo Fisher Scientific) and filtering for low frequency variants (< 1%). Given the clinical suspicion, the binary alignment map (BAM) file was meticulously inspected at the *RBM8A* locus, using Alamut software (Alamut™ Visual Plus, version 1.5.1, SOPHiA GENETICS SA., Saint-Sulpice, Switzerland), to search for further causative variants. The identified variants were confirmed by Sanger sequencing as described above, using custom designed primers (Table S1). HGVS nomenclature was according to the *RBM8A* reference sequence NM_005105.4; LRG_574t1.

Characterization of a novel splice-site variant

RNA from patient F2.II.1 and from controls was extracted from peripheral blood using PerfectPure RNA Blood Kit (5 Prime, Hamburg, Germany) according to the manufacturer's protocol. Resulting purified RNA was converted to cDNA following the protocol for Reverse Transcription SuperScript IV VILO Master MIX, with ezDNA, (Thermo Fisher Scientific, Massachusetts, USA). The cDNA region

corresponding to *RBM8A* exons 2 to 6 was amplified using custom designed primers (Table S1 – cDNA). Amplification products were separated on a 1.5% LE agarose gel (GRiSP Research Solutions, Porto, Portugal), excised, purified with ExoStar 1-step™ and sequenced as described above. The sequencing results were analysed with FinchTV 1.4 software and compared to normal controls.

Supplementary Table S1: Primer sequences designed for amplification and sequencing of genomic DNA (5'UTR and intronic region) and of the cDNA region corresponding to the exons 2 to 6.

	Target	Forward primer	Reverse primer
**gDNA**	5'UTR [c.-21G>A]	GCCTTTGATTGGTCAGCTTGAC	TCCAGTCTTAGTGCCTTCCCG
	intron 4 [c.343-2A>G]	GTCAGACACGCCAAAGAGACAG	AAGGCTGCATGGTCAAATGG
**cDNA**	Exons 2-6	TCACAAACTGAAAGAAAAAGCG	CTCCGGTCTGGACTTCTGC
